# Automatic diagnosis of late-life depression by 3D convolutional neural networks and cross-sample Entropy analysis from resting-state fMRI

**DOI:** 10.1007/s11682-022-00748-0

**Published:** 2022-11-24

**Authors:** Chemin Lin, Shwu-Hua Lee, Chih-Mao Huang, Guan-Yen Chen, Wei Chang, Ho-Ling Liu, Shu-Hang Ng, Tatia Mei-Chun Lee, Shun-Chi Wu

**Affiliations:** 1 Department of Psychiatry, Keelung Chang Gung Memorial Hospital, Keelung City, Taiwan; 2 College of Medicine, Chang Gung University, Taoyuan County, Taiwan; 3 Community Medicine Research Center, Chang Gung Memorial Hospital, Keelung, Keelung, Taiwan; 4 Department of Psychiatry, Linkou Chang Gung Memorial Hospital, Taoyuan County, Taiwan; 5 Department of Biological Science and Technology, National Yang Ming Chiao Tung University, Hsinchu, Taiwan; 6 Department of Engineering and System Science, National Tsing Hua University, Hsinchu, Taiwan; 7 Department of Imaging Physics, University of Texas MD Anderson Cancer Center, Houston, TX USA; 8 Department of Head and Neck Oncology Group, Linkou Chang Gung Memorial Hospital, Chang Gung University, Taoyuan, Taiwan; 9 Department of Diagnostic Radiology, Linkou Chang Gung Memorial Hospital and Chang Gung University, Taoyuan, Taiwan; 10 State Key Laboratory of Brain and Cognitive Sciences, The University of Hong Kong, Hong Kong, Hong Kong; 11 Laboratory of Neuropsychology and Human Neuroscience, The University of Hong Kong, Hong Kong, Hong Kong; 12 Institute of Clinical Neuropsychology, The University of Hong Kong, Hong Kong, Hong Kong

**Keywords:** Cross-sample entropy, Convolutional neural networks, Disease classification, Late-life depression, Machine learning

## Abstract

Resting-state fMRI has been widely used in investigating the pathophysiology of late-life depression (LLD). Unlike the conventional linear approach, cross-sample entropy (CSE) analysis shows the nonlinear property in fMRI signals between brain regions. Moreover, recent advances in deep learning, such as convolutional neural networks (CNNs), provide a timely application for understanding LLD. Accurate and prompt diagnosis is essential in LLD; hence, this study aimed to combine CNN and CSE analysis to discriminate LLD patients and non-depressed comparison older adults based on brain resting-state fMRI signals. Seventy-seven older adults, including 49 patients and 28 comparison older adults, were included for fMRI scans. Three-dimensional CSEs with volumes corresponding to 90 seed regions of interest of each participant were developed and fed into models for disease classification and depression severity prediction. We obtained a diagnostic accuracy > 85% in the superior frontal gyrus (left dorsolateral and right orbital parts), left insula, and right middle occipital gyrus. With a mean root-mean-square error (RMSE) of 2.41, three separate models were required to predict depressive symptoms in the severe, moderate, and mild depression groups. The CSE volumes in the left inferior parietal lobule, left parahippocampal gyrus, and left postcentral gyrus performed best in each respective model. Combined complexity analysis and deep learning algorithms can classify patients with LLD from comparison older adults and predict symptom severity based on fMRI data. Such application can be utilized in precision medicine for disease detection and symptom monitoring in LLD.

**Supplementary Information:**

The online version contains supplementary material available at 10.1007/s11682-022-00748-0.

## Introduction

Late-life depression (LLD) is a prevalent mental disorder among older adults, with incidence ranging from 0.2 to 14.1 per 100 person-years (Büchtemann et al., 2012). LLD is associated with rapid cognitive decline (Chodosh et al., 2007), progression to dementia (Richard et al., 2013), suicide (Conwell et al., 2002), and social burden (Zivin et al., 2013).The development of artificial intelligence (AI) bring an evolutionary change in mental health (Lovejoy, 2019). With accumulated data from electronic health records, brain imaging, wearable devices, and even social media content, AI can predict or classify a variety of mental health illnesses (Graham et al., 2019). The results from AI may validate and complement our knowledge in psychiatry.

Machine learning is a subdiscipline of AI, where algorithms can learn the associations from data and produce predictions on data (i.e., learning from experience) (Jordan & Mitchell, 2015). By using more wide-range statistical models without a priori assumptions, machine learning has been applied in research on LLD. For example, using clinical and structural neuroimaging data, alternating decision trees can predict diagnosis and treatment response in LLD (Patel et al., 2015). Moreover, support-vector clustering can discriminate LLD patients with cognitive impairment from those without such impairment based on the abnormalities of three proteins (Apo AI, IL-12, and stem cell factor) (Diniz et al., 2015). These studies suggest that machine learning can handle complex behavioral and biological data in LLD.

The implementation of machine learning in neuroimaging analysis is appropriate as algorithms can manage datasets with more variables than observations (Janssen et al., 2018). Traditional resting-state functional connectivity, by measuring the temporal correlation between two anatomically separate brain regions at rest, has been widely used in the research of LLD (Tadayonnejad & Ajilore, 2014). However, such static analysis ignores the dynamic and nonlinear information in this typical 5–10-minute resting-state scan (Xie et al., 2008). Entropy analysis (Richman & Moorman, 2000), a way to measure randomness and predictability in the data, has been adopted to capture the dynamic and complexity of the temporal fluctuation in the resting brain (Wang et al., 2018). However, uncorrelated random signals (i.e., white noise) have high entropy value but are not complex at all, limiting the use of traditional entropy analysis. Therefore, multiscale sample entropy (MSE) analysis is developed where the complexity of the system is analyzed under different scales of the time series (Jiang et al., 2011). MSE analysis can better capture the physiological changes in biological system with the applications from cardiac arrhythmia to aging process (Costa et al., 2005). Using multiscale sample entropy analysis in the analysis of the resting-state fMRI, we have recently shown entropy differences in certain brain regions in LLD with particularly the adaptive increase of entropy in the left frontoparietal network (Lin et al., 2019). Complimentary to connectivity analysis, entropy analysis can provide additional insights on how the complexity of brain signal changes (McDonough & Nashiro, 2014). Given that mental disorders were hypothesized to be associated with complexity changes in the brain (Takahashi, 2013; Yang & Tsai, 2013), the combination of entropy analysis and machine learning in resting-state fMRI could provide innovative features in LLD.

By extending our previous work, the current study adopted cross-sample entropy (CSE) analysis, which measures the similarity between two time series (i.e., the relation of the complexity between two brain regions of interest [ROIs] in this case). Moreover, we constructed a three-dimensional (3D) CSE volume for each subject (Wang et al., 2014). Yielding to the high computational demand, Convolutional Neural Networks (CNN) was used. Unlike traditional machine learning, deep learning obtains features in the data by automatic learning through sets of neural networks. CNN, initially inspired by the primate visual system, is a type of neural network that excels particularly in 3D image recognition and classification (Koppe et al., 2021; Segato et al., 2020). To date, CNN has been applied in resting-state fMRI data to diagnose Alzheimer’s disease (Duc et al., 2020), attention deficit hyperactivity disorder (Ariyarathne et al., 2020), and schizophrenia (Qureshi et al., 2019). Thus, this study aimed to investigate which brain region could predict LLD diagnosis and depression severity most accurately using 3D-CNN with the input of CSE volumes of the resting-state fMRI signal. We expect that our result will further the use of machine learning in fMRI analysis, which helps in understanding the brain’s functional architecture in LLD.

## Methods

### Participants

Patients recruited from psychiatric outpatient service were at least 60 years old with at least one major depression episode, based on the DSM-5 diagnosis, after age of 60 (regardless of the age of the 1st depression onset, inclusive of early-onset and late-onset depression). Depression severity was measured using the 17-item Hamilton depression rating scale (HAM-D) (Hamilton, 1967). We recruited patients who were still in active depressive phase by only including those who had HAM-D score above 7 (i.e., not remitted). The patients were allowed to keep their psychotropics during the study due to ethical reasons, with the dosage maintained for at least 2 months before the MRI scan. Except for anxiety disorder, no other major Axis I psychiatric disorder was diagnosed. All the participants had no history of significant head trauma, stroke, major neurocognitive decline, Parkinson’s disease, thyroid dysfunction, or other major neurological disorders and had a minimum score of 24 on the Mini-Mental State Examination (Folstein et al., 1983). Cognitively normal older adults without any major Axis I psychiatric diagnosis were recruited by advertisements in community centers. Patients in the LLD group were allowed to keep their psychotropics during the fMRI scan for ethical reason. However, their medication had been unchanged for at least two months prior the scan, and we also calculated the medication load for each participant according to Antidepressant Treatment History Form ATHF-modified (Sackeim, 2001). The study protocols have been approved by the institutional review board of the Chang Gung Medical Foundation (IRB No. 201202970B0C601). All the study participants provided written informed consent. The participants were merged from two consecutive projects in order to gain adequate power in the model building (Lin et al., 2021; Lin et al., 2019).

### Data acquisition

We collected our MRI data using an eight-channel head coil on a 3T MRI scanner (Discovery MR750, GE Healthcare, Milwaukee, WI). Participants were asked to keep their eyes closed, not to think of anything, and not to fall asleep during the scan. The resting-state functional MRI data were collected using a T2*-weighted gradient-echo echo-planar imaging sequence with the following parameters: repetition time (TR), 2,000 ms; echo time (TE), 30 ms; flip angle, 90°; number of slices, 36; in-plane matrix size, 64 × 64; and slice thickness, 4 mm. A total of 180 dynamic volumes were acquired for each subject. The T1-weighted structural imaging was acquired with TR, 8 ms; TE, 3 ms; flip angle, 12°; FOV, 250 × 250 mm^2^; voxel size, 0.98 × 0.98 × 1 mm^3^; and slice number, 160.

### Image preprocessing

The preprocessing included the following steps: slice-timing correction, motion correction by realigning images to the first volume and removing those images showing 2-mm axial displacement and 2° in rotation angle, normalization and deformation to Montreal Neurological Institute template, and reslicing to 2 × 2 × 2 isotropic voxel dimensions. These procedures were implemented using SPM12 (Statistical Parametric Mapping, http://www.fil.ion.ucl.ac.uk/spm/). Further preprocessing steps used the REST toolbox (http://restfmri.net/forum/REST_V1.8). At each voxel, the time series were detrended and bandpass-filtered (frequencies between 0.01 and 0.08 Hz). The time courses for various covariates (white matter, cerebrospinal fluid, and six motion parameters for head movement) were extracted and regressed out as nuisance regressors for eliminating potential impacts of physiological artifacts. Finally, the gray matter of the brain was divided into 90 ROIs according to the Automated Anatomical Labeling (AAL) (Tzourio-Mazoyer et al., 2002). The data time series of all voxels in an ROI were averaged. The brain networks were visualized using the BrainNet Viewer toolbox (http://www.nitrc.org/projects/bnv/) (Xia et al., 2013).

### Cross-sample entropy

CSE is a measure to assess the degree of asynchrony or dissimilarity of two data series. It remains relatively consistent for conditions where the cross-approximate entropy does not and exhibits no direction dependence (Gómez et al., 2009; Richman & Moorman, 2000). With this measure, the functional connectivity between the brain regions and complexity of their data series can be simultaneously revealed (Zhang et al., 2007). Given two normalized data series $\mathbf{x}={\text{ }}[{x_1},{\text{ }}{x_2},...,{\text{ }}{x_L}]$ and $\mathbf{y}={\text{ }}[{y_1},{\text{ }}{{\text{y}}_2},...,{\text{ }}{{\text{y}}_L}]$, their CSE is defined as

1 $${\text{CSE}}(m,r,L)= - \ln \frac{{p_{{}}^{{m+1}}}}{{p_{{}}^{m}}},$$

where $p_{{}}^{l}=\sum\limits_{{i=1}}^{{L - l}} {p_{i}^{l}} /(L - l)$ with *l* (*m* or *m* + 1 in (1) with *m* = 2 in this study) being the length of two sub-vectors ${\mathbf{y}_l}(j)=[{y_j},{\text{ }}{y_{j+1}},...,{{\text{y}}_{j+l - 1}}]$ and ${\mathbf{x}_l}(i)=[{x_i},{\text{ }}{x_{i+1}},...,{\text{ }}{x_{i+l - 1}}]$ from data series **y** and **x**, respectively.$p_{i}^{l}=n_{i}^{l}/(L - l)$ and $n_{i}^{l}$ are the probability and number of vectors that any *l*-point sub-vector ${\mathbf{y}_l}(j)$ in the data series **y** that matches the *l*-point sub-vector ${\mathbf{x}_l}(i)$ in the data series **x**, respectively. The indices *i* and *j* vary from 1 to *L* - *l*. A match with a tolerance *r* is defined to be $d\left[ {{\mathbf{x}_l}(i),{\text{ }}{\mathbf{y}_l}(j)} \right]<r$ with

2 $$d\left[ {{\mathbf{x}_l}(i),{\text{ }}{\mathbf{y}_l}(j)} \right]=\hbox{max} \left\{ {\left| {{x_{i+k}} - {y_{j+k}}} \right|:0 \leqslant k \leqslant l - 1} \right\},$$

which is the maximum difference between the components of ${\mathbf{x}_l}(i)$ and ${\mathbf{y}_l}(j)$. The tolerance parameter *r* is determined when the two sub-vectors match, which was set to be 0.6 in this study.

**Fig. 1:**
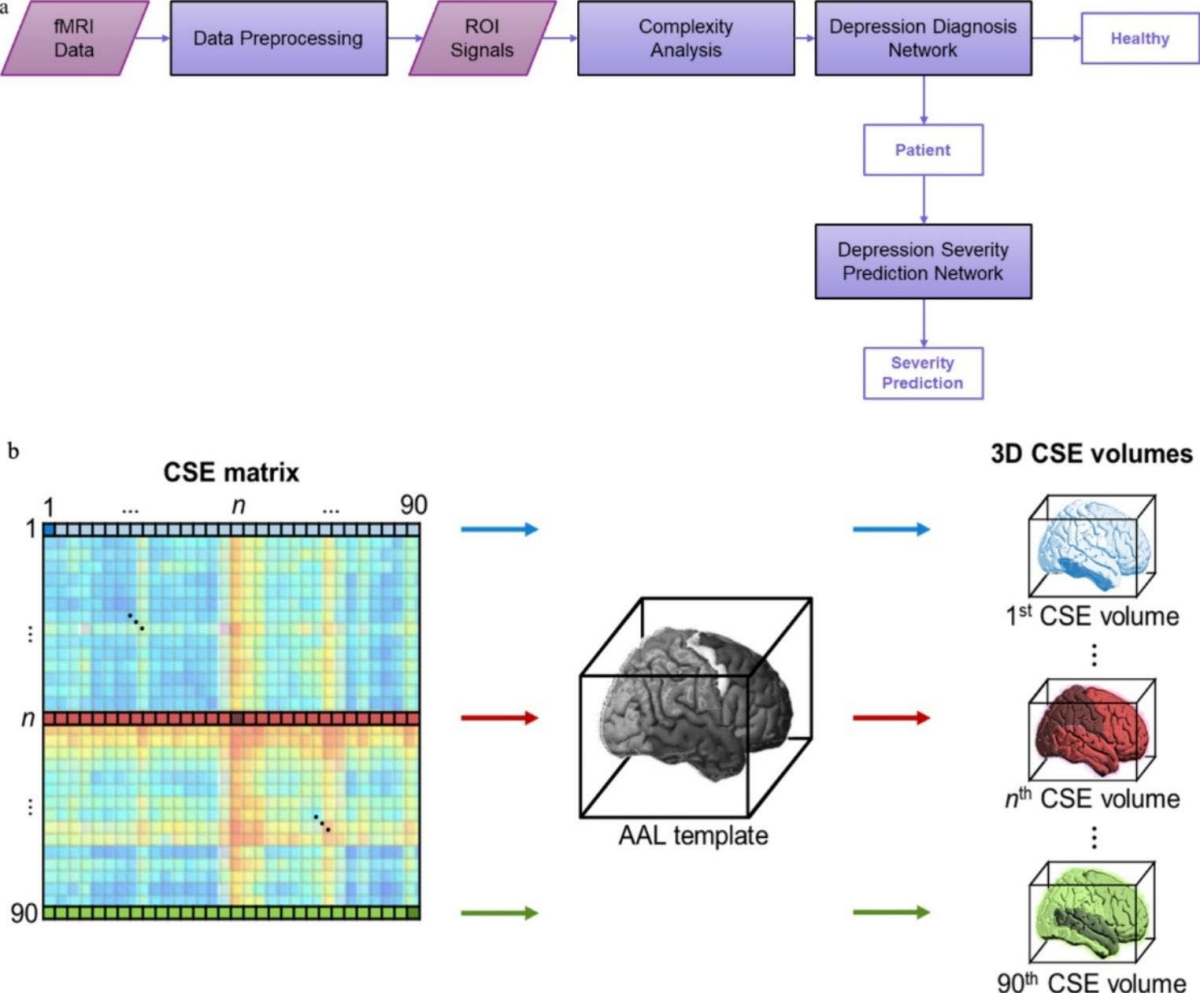
(a) The proposed scheme for depression diagnosis and symptom severity prediction. (b) The construction of 3-D CSE volume by mapping the CSE value in the seed ROI and the remaining ROIs in the brain

The CSE matrix in Fig. 1b is a 90 × 90 square matrix containing CSE values between the AAL ROIs. The calculation of these CSE values starts from pairing the data time series of the 90 ROIs and is obtained using (1). Note that the CSE matrix is symmetric, with its main diagonal containing the sample entropy of each ROI (i.e., the CSE of each ROI with itself). Furthermore, the *i*-th column of the CSE map contains the CSE values of the 90 ROIs with respect to the *i*-th ROI. Assigning each entry of the *i*-th column of the CSE map to all the voxels of the corresponding AAL ROI, we can construct a 3D CSE volume that uses the *i*-th ROI as the “seed.” The resulting 3D CSE volume has a size of 91$\times$109$\times$91, which serves as the input to the proposed network models. Having a large enough dataset is always essential for the performance of a deep learning model. Thus, a data augmentation method is incorporated before calculating the CSE values. More specifically, for a data time series of length *N* (i.e., 180 in this study), it is partitioned into three segments of length *N*/2 overlapped with *N*/4 so that the first and third segments are the first and last halves of the original data time series. The second segment comprises the last and first halves of the first and third segments. By doing so, we triple the data and thus the 3D CSE volumes for each subject. Finally, since the CSE matrix encodes only temporal information about brain activity, combining it with the 3D brain structure provides spatial-temporal details on brain interaction. We also center and scale each entry in a CSE matrix before constructing its 3D CSE volumes according to the mean and standard deviation of the normal subjects’ CSE matrices.

### Convolutional neural networks

The classification model for depression diagnosis comprises one average-pooling, three convolution, two max-pooling, two batch normalization, one flatten, and four fully connected layers. The architecture starts with the average-pooling layer. Two of the three convolution layers are followed, each with the max-pooling and batch normalization layers intervened. There is no consensus on the arrangement for the pooling and batch normalization layers, and one may try out different arrangements for “smooth” training experiences (Ioffe & Szegedy, 2015). We noted that they might be used interchangeably for various classification and regression purposes (Hasani & Khotanlou, 2019; Herent et al., 2018; Khvostikov et al., 2018). Then, the third convolutional layers are connected, whose output is fed to the four successive fully connected layers. The input to the network is a data block of size 91 × 109 × 91 (i.e., the CSE volume of a specific seed ROI). The convolution layers have 32, 64, and 128 filters of sizes 3 × 3 × 3, 3 × 3 × 3, and 2 × 2 × 2, respectively. The filter sizes for the average-pooling and the two max-pooling layers are all 2 × 2 × 2. The number of neurons is 500 in the first three fully connected layers and 2 in the last one. The two severity level classification networks share the same architecture. Although we defined the HAM-D scales of > 13, 11–13, and 8–10 as severe, moderate, and mild scale levels, respectively, we trained the model of each level with HAM-D scales lying in the ranges of > 11, 9–15, and 8–12, respectively. The overlapped ranges of the HAM-D scale offer fault-tolerance for the networks, which is crucial to provide consistent results for HAM-D scale prediction.

The proposed regression network model for HAM-D scale prediction resembles the above classification network, except that it comprises seven fully connected layers in series instead of four and that the batch normalization layers are applied before max-pooling layers. The convolution layers have 32, 64, and 128 filters, and all are of sizes 3 × 3 × 3. The filter sizes for the average-pooling and the two max-pooling layers are 2 × 2 × 2 and 3 × 3 × 3, respectively. The number of neurons is 1028 in the first three fully connected layers, 512 in the subsequent three layers, and 1 in the last layer. Note that the regression networks conditioned with the CSE volumes constructed from different seed ROIs show the same architecture.

All the above-mentioned conditioned layers are followed by the ReLU activation function, except for the last fully connected layer, where the softmax function is used in the classification network and the linear function is used in the regression network. The architectures of the network models for classification and regression are illustrated in Supplementary Fig. 2.

### Model training

We retained most participants for the training dataset to ensure enough data covering each HAM-D scale for model training, resulting in 51, 7, and 13 participants in the training, validation, and testing sets for depression diagnosis and 29, 10, and 10 participants in the corresponding sets for severity scale prediction. The concept for data splitting is to ensure that data set contains approximately the same number of observations in each participant group and each HAM-D scale. We used the categorical cross-entropy loss for classification and the mean squared error for regression during model training; both were optimized using the adaptive moment estimation optimizer with the epoch size of 200 and the batch size of 32. The learning rate was set as in all the classification models in Depression Diagnosis Network (DDN) and the first severity classification network in Depression Severity Prediction Network (DSPN). For the remaining classification network and regression networks, the learning rate was 1 × 10^− 4^. The training procedure was stopped if the loss did not improve within 60 epochs and 20 epochs in the classification and regression models, respectively. Early stopping was utilized to avoid overfitting (Orr & Müller, 2003). The validation dataset provides a basis to evaluate the model trained on the training dataset. The prediction error on the training dataset will decrease, whereas the error on the validation set will first decrease and then increase. The early stopping point occurs when the error on the validation set is the lowest. Here, the network weights provide the best generalization, and the training will be stopped since an increase in the error on the validation dataset is a sign of overfitting to the training dataset. The models were trained in TensorFlow 1.13.1 with the CUDA 9.2 Toolkit and cuDNN v7.6.0 on the computing platform: ASUS ESC8000 G4 server system with Intel Xeon CPU, GeForce RTX 2080 Ti, and 192 GB RAM.

### Proposed scheme

The flow of the proposed scheme for depression diagnosis and severity scale prediction is depicted in Fig. 1a (In Supplementary Fig. 1, a detailed system diagram of the proposed scheme is provided.) In this scheme, the DDN is used to determine whether the subject under test is depressed. For those determined to be depressed, the HAM-D scores are predicted through the DSPN. At the cores of these two networks is the CNNs for different purposes, with their inputs being the three-dimensional (3D) CSE volumes (Chen et al., 2020; Richman & Moorman, 2000; Zhang et al., 2007). For a given seed ROI, its 3D CSE volume is constructed by mapping the CSE values between the data time series of this seed ROI and those of the remaining ROIs in the brain (Chen et al., 2020), as shown in Fig. 1b.

With the influence of learning strategies (Sagi & Rokach, 2018), the DDN integrates across different seed ROI selections to better determine whether the subject under test is depressed than that achieved by any of the constituent ROIs alone. Thus, without a priori selection of a particular ROI, we hypothesized that the DDN can benefit from integrating different ROI selections. The final decision made by the DDN is obtained through polling the results from the classification models trained on the 3D CSE volumes calculated with different seeds, providing comprehensive investigations of different 3D CSE volumes in depression diagnosis.

In DSPN, not all CSE volumes of different seed ROIs are used for severity scale prediction. Three datasets are involved in deep learning application. The training dataset contains the sample of data used to fit or train the network model. The validation dataset provides the sample of data used to obtain an unbiased estimate of the generalization error of the model acquired from the training set while tuning model hyperparameters, which avoids model overfitting. These two datasets will be considered during the process of model training. Lastly, the test dataset has the sample of data used to provide an unbiased evaluation of a final model fit on the training and validation datasets. The role of any sample in this dataset mimics a future sample we will encounter in practice. With this in mind, to select the CSE volumes for HAM-D scale prediction, we only retain those whose resulting classification models in DDN achieve 85% accuracy during model validation. This means that no information from the CSE volumes that will be used to evaluate the performance of the final network model will be used in this selection. Moreover, the ROIs (49 in this study) used as the seeds for computing the CSE volumes that led to better diagnosis accuracy may induce insights that are crucial in discriminating depressed patients from normal subjects and will be further explored. The accuracy for node here means the percentage of correct classification in disease diagnosis or symptoms prediction.

To predict the HAM-D scale of a depressed subject, we adopt a two-stage strategy in DSPN. A depressed subject is first classified to be of a severe, moderate, or mild scale level. Here we define severe scale level as HAM-D scales > 13, moderate scale as between 11 and 13, and mild scale as ≤ 10. Two severity level classification networks are used to achieve this classification task. The first network, composed of *m* (again 49 in this study) independent weak classifiers, categorizes the depressed subjects into high- or low-scale levels. Those in the low-scale levels are further divided into moderate or mild scale levels using the second severity classification network which comprises merely one classifier. Each of the *m* CSE volumes of the subject is fed into this network to predict the severity level, and all the determined levels are then polled to decide the most likely severity level of that subject. Finally, we predict the HAM-D scale of the depressed subject using the regression network according to which severity level the subject belongs to, enabling precise HAM-D scale prediction. Moreover, this network consists of merely one regression model for each severity level, and the predicted HAM-D scales obtained by feeding different CSE volumes to the regression model are averaged and rounded to the nearest integer to obtain the ultimate HAM-D scale for that subject.

## Results

We enrolled 77 participants, including 49 late-life depressed patients and 28 non-depressed comparison older adults, with a mean age of 67.7 ± 6.0 years. The LLD group had lower education level and higher HAMD score than the comparison group. The average onset of the first depressive episode was 50.4 ± 11.6 with a mean 3.3 ± 2.9 episodes in the LLD group. Also, all the patients in the LLD group were not remitted from their current depressive episode with a mean HAM-D score of 13.3 ± 3.6. Thus, only five patients were not under antidepressant, while the rest all received antidepressants during the study period, including 19 under selective serotonin reuptake inhibitors, eight under mirtazapine, seven under serotonin and norepinephrine reuptake inhibitors, six under agomelatine, and four under bupropion. No group difference was found in age, sex and MMSE score (Table 1).

**Table 1:** Demographic data and between group comparisons

	Non-depressed older adults	LLD	Statistics
	(n = 28)	(n = 49)	
Age	69.4 ± 5.8	66.8 ± 6.0	t = 2.0, df = 75
Sex, (M/F)	11/17	18/31	Chi = 0.05, df = 1
Education	11.4 ± 4.2	8.2 ± 2.9	t = -3.6 ***, df = 75
MMSE	28.5 ± 1.1	28.2 ± 1.5	t = -1.0, df = 75
HAMD	3.4 ± 2.4	13.3 ± 3.6	t = 13.1***, df = 75

HAMD, 17-item Hamilton Depression Scale; MMSE, Mini-mental Status Examination; *p* < 0.001, df = degree of freedom

**Fig. 2:**
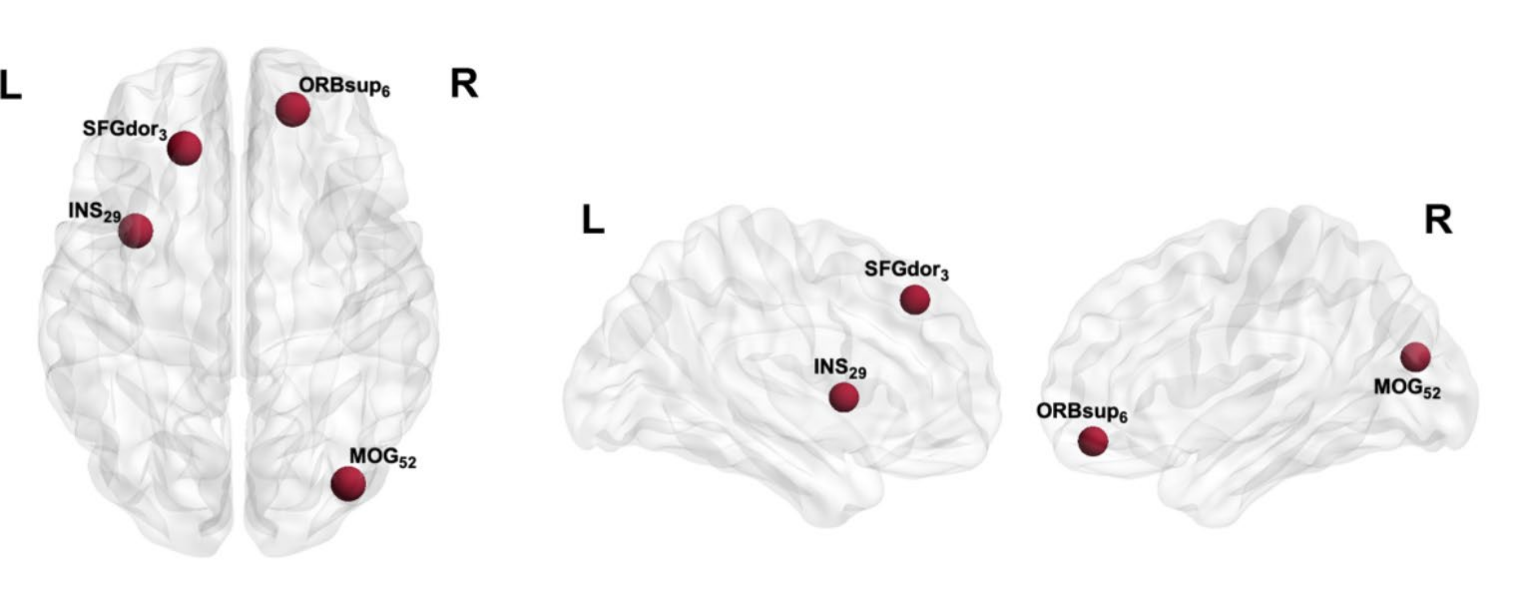
Nodes where CSE volume achieved accuracy rate above 85% in classification model. SFGdor, dorsal superior frontal gyrus; INS, insula; ORBsup, superior frontal gyrus, orbital part; MOG, middle occipital gyrus

In the testing data set, the proposed diagnostic network can correctly classify all the 13 participants. Four ROIs attained accuracy rate above 85% in classification, including the superior frontal gyrus (left dorsolateral and right orbital parts), left insula, and right middle occipital gyrus (Fig. 2). A total of 20 ROIs reached an 80% accuracy rate (Supplementary Fig. 3).

**Fig. 3:**
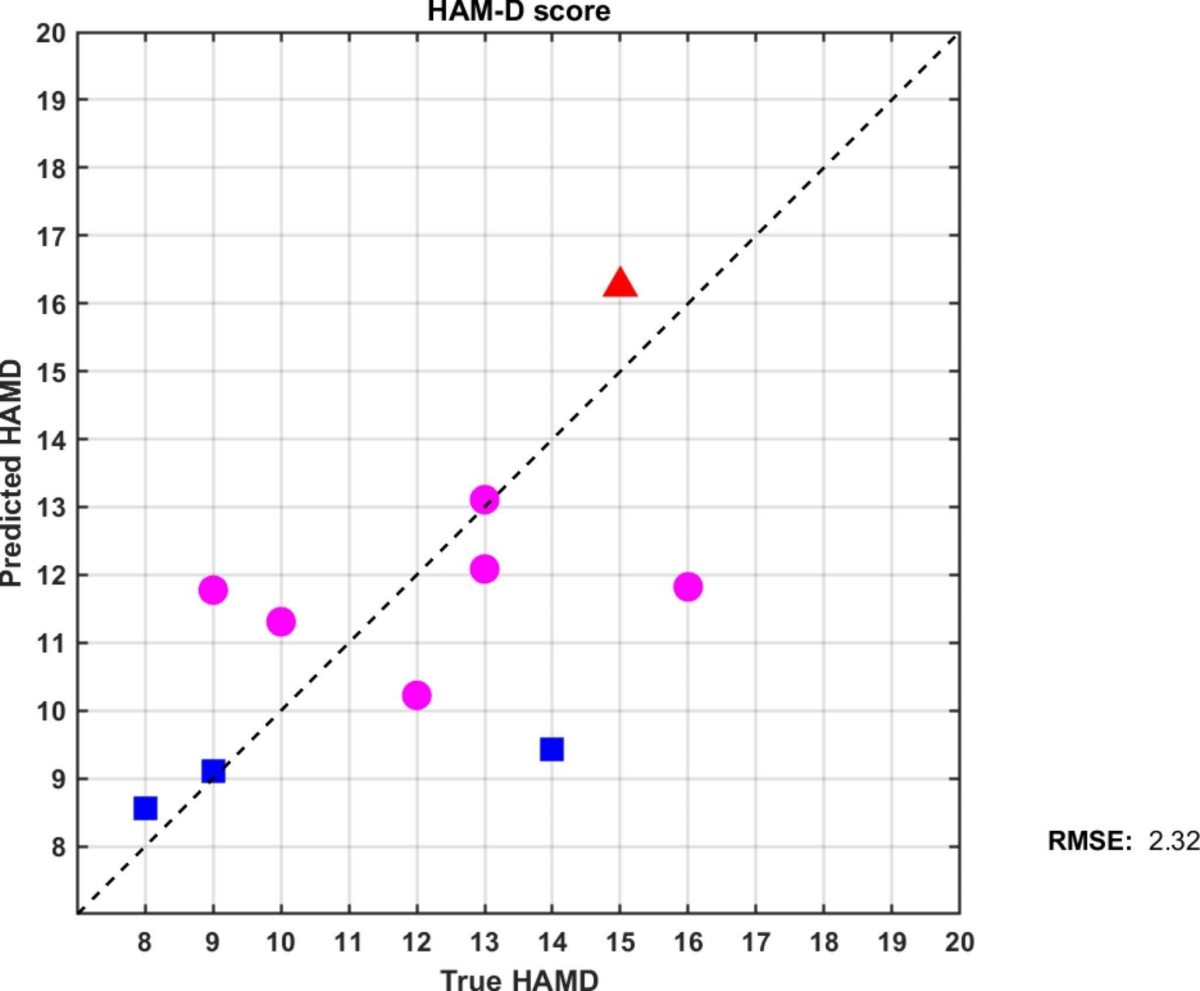
The root-mean-square error (RMSE) between true and predicted HAM-D scales obtained by our proposed scheme for the testing data (RMSE = 2.41).

For DSPN, participants were allocated to three corresponding regression models based on symptom severity. In the 10 testing participants, there were 3 the in severe, 3 in the moderate, and 4 in the mild depression groups. Since we intentionally trained these three prediction models with overlapped cut-off points to allow some fault-tolerance, three subjects were allocated to the wrong models. The root-mean-square error (RMSE) for predicting the HAM-D scales of the 10 subjects was 2.41 (Fig. 3). In post hoc, we manually allocated these three patients into the right models; the RMSE could be reduced to 1.48. This indicated that these prediction models provide good HAM-D scale prediction under appropriate regression models. In each of the three models, we ranked all 90 ROIs based on the RMSE value shown in Supplementary Fig. 4. In the regression model of the severe depression group, the CSE volume in the left inferior parietal lobule performed best with an RMSE of 1.53 in the regression model of the severe depression group, left parahippocampal gyrus with an RMSE of 1.66 in the moderate group, and left postcentral gyrus with an RMSE of 0.50 in the mild group (Supplement Fig. 4a ~ c).

## Discussion

Based on the CSE analysis of resting-state fMRI data, we demonstrated that 3D-CNN machine learning algorithm could classify late-life depressed patients from comparison group and predict depression severity. The 3D-CSE volume in regions such as the superior frontal gyrus (left dorsolateral and right orbital parts), left insula, and right middle occipital gyrus, could discriminate LLD from the comparison group with an accuracy rate above 85%. Regions in the left inferior parietal lobule, left parahippocampal gyrus, and left postcentral gyrus best predict HAM-D score in severe, moderate, and mild depression groups, respectively. Combined entropy analysis and 3D CNN approach can reveal complementary and additional brain features in LLD.

Two nodes in the superior frontal gyrus were found to have high predictive power, classifying LLD from non-depressed older adults. Brain atrophy in the superior frontal gyrus has been found in a meta-analysis on LLD (Boccia et al., 2015). In particular, our finding of the dorsolateral part of the prefrontal gyrus (DLPFC) is consistent with prior research showing persistent functional connectivity decrease in DLPFC even after treatment (Aizenstein et al., 2009). The anterior and posterior subdivisions of the dorsolateral SFG correlated with the default mode network (DMN) and other regions involved in cognitive control, respectively (Li et al., 2013), which are both implicated in the pathophysiology of depression. Moreover, the orbital SFG is implicated in depression, whose nodal efficiency, which measures the community efficiency in a network, has been found to be positively related to depression severity in diffusion tensor imaging study (Qin et al., 2014). Orbital SFC is referred to as medial orbital frontal gyrus (OFC) under newer brain parcellations (Heather Hsu et al., 2020; Rolls et al., 2015). Decreased activity in the medial OFC is associated with decreased reward experience and depressive symptoms, especially anhedonia and apathy, which are two common symptoms of LLD (Rolls, 2019). The insula shares a similar relationship with the OFC (Ghaziri et al., 2017) and is a key node in the salience network. Decreased salience network (insula) connectivity with left executive control network (ECN) has been found to be correlated with executive dysfunction in LLD, suggesting insula’s crucial role in task-switching function (Li et al., 2017). Furthermore, we also found the right middle occipital gyrus (MOG) to be highly predictive of LLD. A past study reported decreased functional connectivity in the right MOG within ECN in patients remitted from LLD (Karim et al., 2017). In LLD, increased hyperconnectivity has been found in the DMN and auditory and visual networks (Eyre et al., 2016), highlighting the significance of the nodes in occipital cortex in LLD.

Regarding depression severity prediction, we could not obtain acceptable accuracy rate by using only one model. Instead, three separate models were required for the high, moderate, and low depression groups. Also, the anchor points for our categorization into three groups were different from the conventional grouping to define mild (8–16), moderate (17–23), and severe depression (≥ 24) (Zimmerman et al., 2013). The conventional categorization performed poorly in our sample. However, the nature of the model formation is data-driven and reflects the range of depression severity in our sample (range in HAMD: 8–20). The ROIs derived from these three models were different from each other, offering neurobiological evidence to this categorization and challenging the traditional cutoffs to define depression severity categories. For example, the left inferior parietal lobule and left parahippocampal gyrus were the nodes performing best to predict symptom severity in high and moderate depression groups, respectively. Both nodes belong to the DMN, where the heightened activity is the central pathophysiology of depression (Sheline et al., 2009). In contrast, the CSE in the left postcentral gyrus can best predict the depression severity in the mild depression group. The white matter hyperintensity (WMH)-related structural dysconnectivity in postcentral gyrus was associated with slower processing speed and reduced attentional set-shifting in LLD (Respino et al., 2019). Moreover, increased functional connectivity between the right postcentral gyrus and ECN was determined after treatment of LLD (Karim et al., 2017). All these studies suggest that the structural and functional integrity in postcentral gyrus is associated with the executive dysfunction in LLD. Thus, nodes from the DMN demonstrate higher predictive power in those of more severe depression, whereas nodes related to cognitive function are for those of milder depression.

Unlike traditional linear functional connectivity analysis, we adopted an alternative nonlinear approach to analyze the functional connectivity of distinct brain regions (Hu & Shi, 2006). CSE is used to detect the asynchrony of the signals between two brain regions, reflecting the complexity of local and distributed information processing within the brain. Entropy is also applied to understand the consciousness of the brain (Carhart-Harris, 2018), where a certain range of the entropy is required for the brain to maintain its function (i.e., a state called “criticality”, a critical range between order and disorder). In addition to self-organized criticality, scale-free is another attribute of the brain dynamic network (Hesse & Gross, 2014; Massobrio et al., 2015). The scale-free brain network follows the power-law distribution, resulting in only a few “hubs” in the brain with multiple connections with other regions and endowing the brain network resilience toward random attack (Aerts et al., 2016; Albert et al., 2000). This scale-free property of the brain network could be demonstrated by CSE analysis (Pritchard et al., 2014). Thus, these prior studies have suggested entropy analysis suitable to capture the scale-free and criticality features of the brain network. The notion of entropy is also implicated in the understanding the formation of depression, a status regarded as one’s adaptive behavior to minimize the uncertainty in life by reducing the entropy of the sensory and physical states (Badcock et al., 2017).

Regarding the limitations, the sample size was small to cover each HAM-D scale for model training. Although the independent testing dataset used in this study can still measure the generalization performance for unknown future cases, data from more samples for model training are beneficial to model generalization. Furthermore, the small number in our testing data is noteworthy as low testing data could lead to misestimation of model performance (Flint et al., 2021). The polls of few subjects were won by a margin of only 10% of the 90 seed ROIs’ votes, and those in the margin may lead to potential failure in depression diagnosis. Moreover, our modification using overlapped cut-off points in HAM-D presents fault-tolerance in the depression symptoms prediction network. However, further studies including participants with a larger variance in depression symptoms are warranted. Third, we only used resting-state fMRI data. Future studies incorporating multi-modal imaging measures may yield superior model performance (Patel et al., 2015). One study did attempt to employ 3D convolutional and recurrent neural networks on structural and functional MRI data, however, with the receiver operating characteristic curve score reaching only 0.73 (Pominova et al., 2018). To date, numerous machine learning approaches have been applied extensively to neuroimaging data to classify depression (Gao et al., 2018), where resting-state fMRI appeared to provide the highest accuracy rate compared to other neuroimaging modalities. Last but not least, we notice the group difference in education level and medication the use of antidepressant in the group of LLD. Speculatively, integrating these variables in the model may augment the model performance. However, our aim of the study is to develop an automatic classification model based solely on resting-state fMRI data without relying on the collection of demographic data or clinical variables. Also, it has been found that antidepressant treatment can obscure or reverse the brain changes due to depression (Zhuo et al., 2019). Were it not for the continuing antidepressants use in the LLD group, our proposed CSE-based CNN model could provide more salient features with higher accuracy rate in the model. Nevertheless, future validation is warranted in treatment naïve or medication-free older adults with LLD.

## Conclusion

In conclusion, our results also highlight the importance to include both the temporal (i.e., the multiscale entropy analysis) and the spatial (i.e., the 3D approach) features of the fMRI data in order to construct an effective model. Our new machine learning approach for neuroimaging data provides new insights into understanding the neurobiology of LLD.

## Electronic supplementary material

Below is the link to the electronic supplementary material.

Supplementary Material 1

Supplementary Material 2

Supplementary Material 3
