# Supplementary material for: Automatic diagnosis of late-life depression by 3D convolutional neural networks and cross-sample Entropy analysis from resting-state fMRI
Source: Brain Imaging Behav. 2022 Nov 24;17(1):125–35. doi: 10.1007/s11682-022-00748-0 (PMC9922223; doi:10.1007/s11682-022-00748-0)
Supplement: Supplementary file 1 — Supplementary Material 1 [file 11682_2022_748_MOESM1_ESM.docx]

Supplementary Figure 1. (a) System diagram of the proposed scheme for depression diagnosis and the proposed two-stage scheme for severity prediction. (b) Structure diagram for HAM-D scale estimation.

**
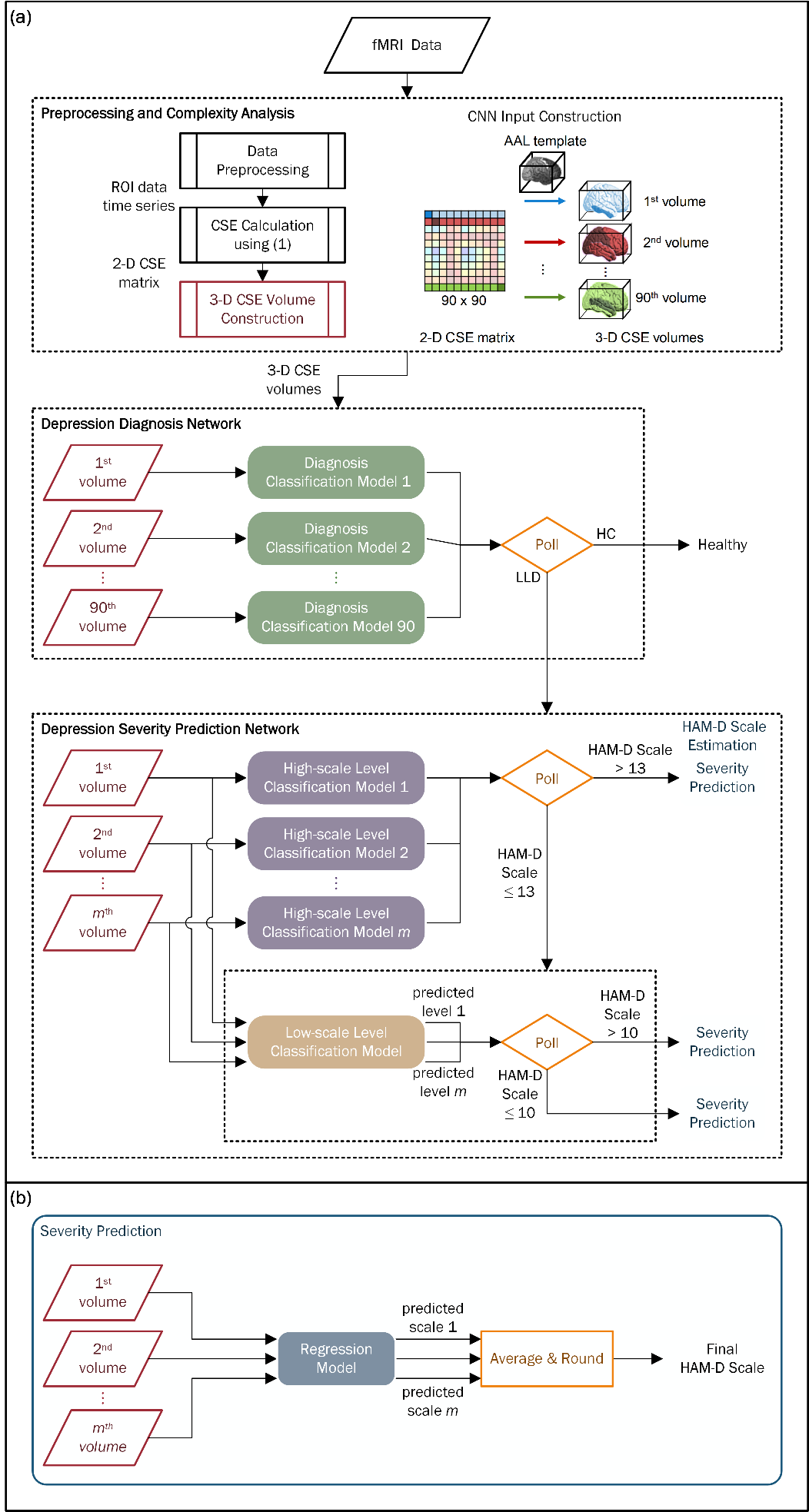
**

Supplementary Figure 2. The architectures of the proposed network models for classification and regression.


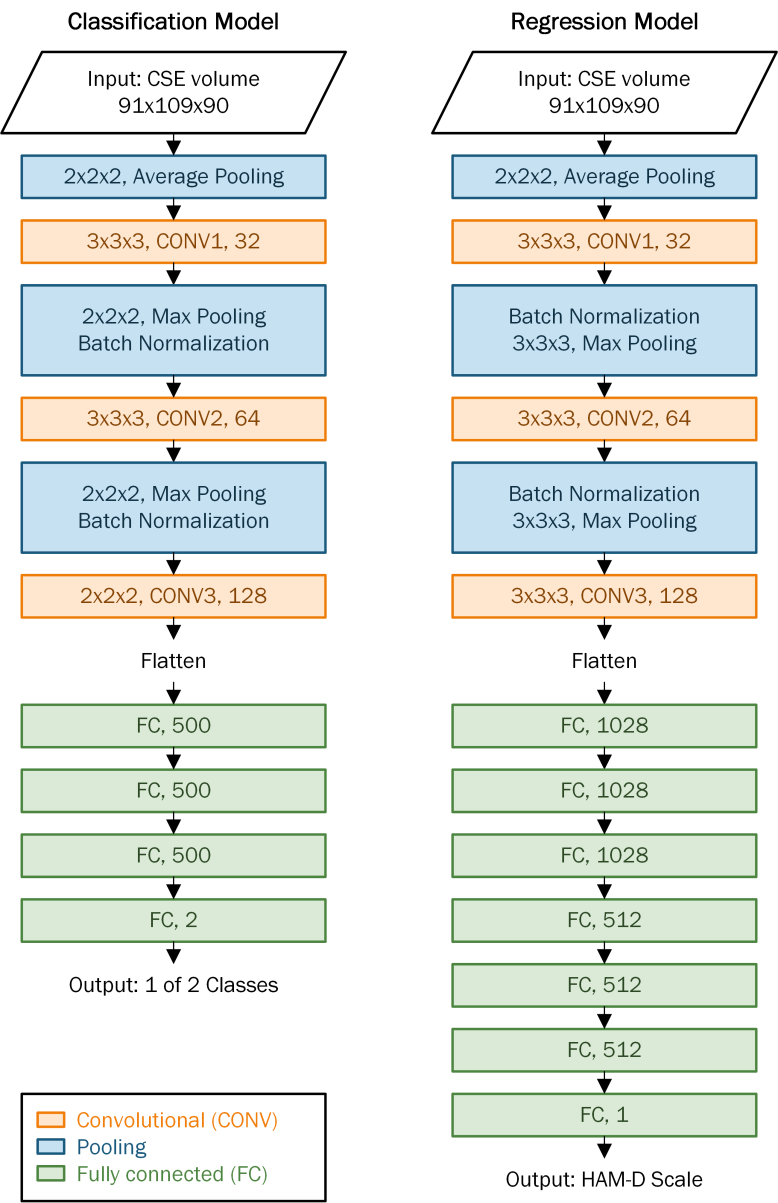


Supplementary Figure 3. Nodes where CSE volume achieved accuracy rate above 80% in classification model (a total of 20 ROIs).


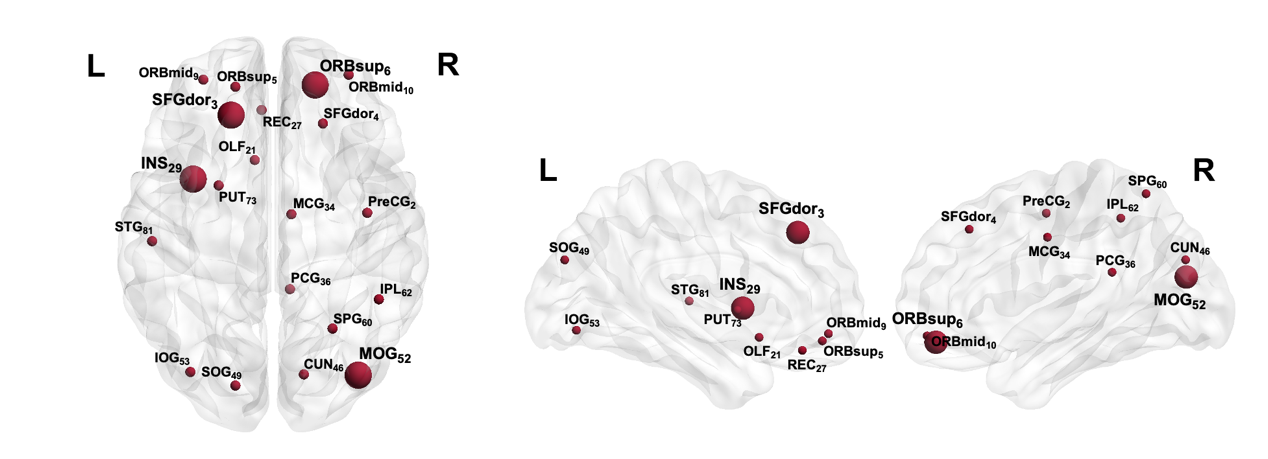


Supplementary Figure 4. The ROIs whose CSE volumes best predicting HAM-D scores are ranked based on the performance (i.e., the score of root-mean-square error [RMSE] in the model). For brevity, we rendered the ten ROIs with the lowest RSME in severe (a), moderate (b) and mild (c) depression groups respectively.

to predicting the HAM-D scales among


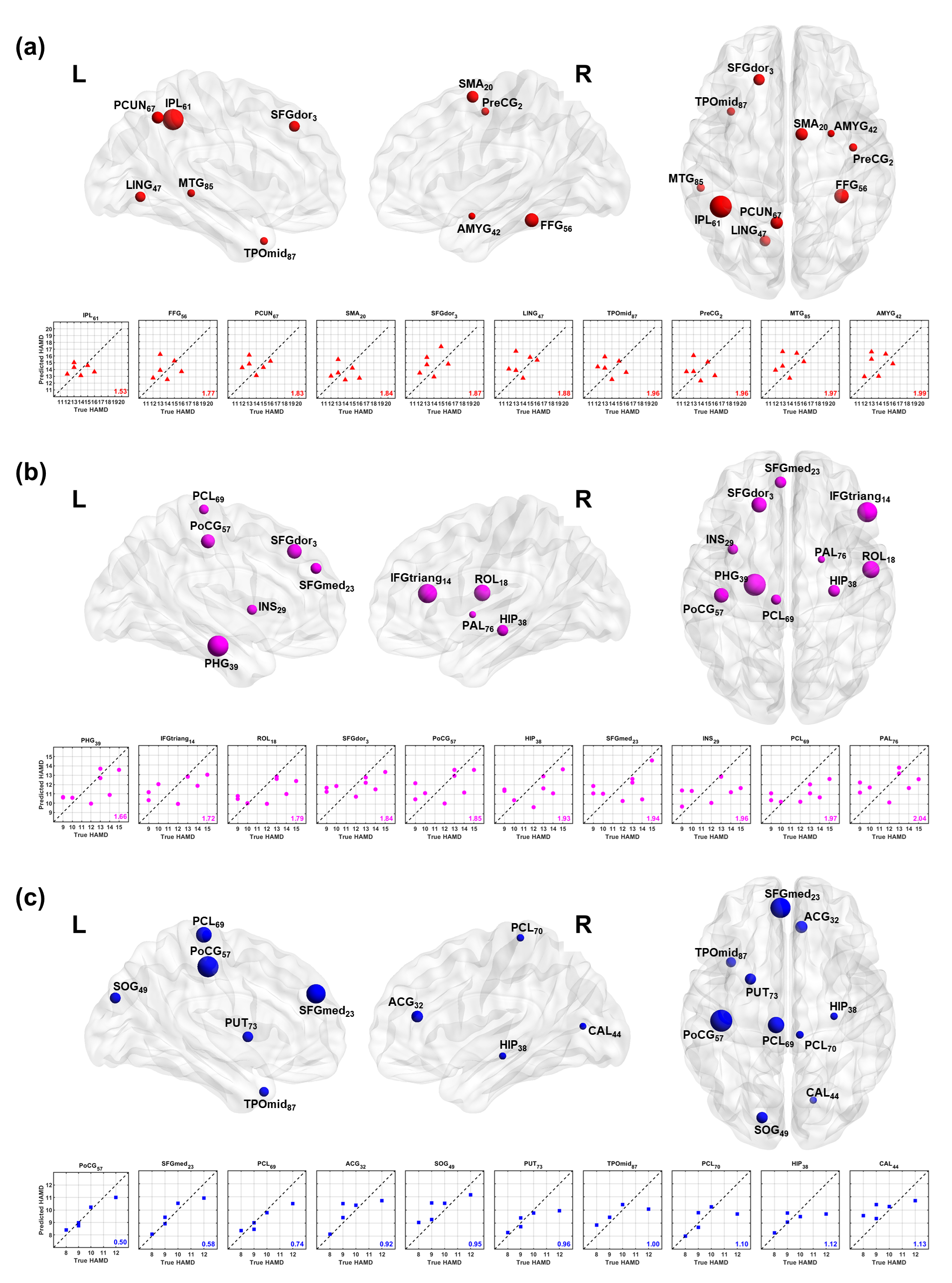


Reference:

Gómez, C., Hornero, R., Abásolo, D., Fernández, A., Escudero, J., 2009. Analysis of MEG background activity in Alzheimer’s disease using nonlinear methods and ANFIS. Annals of Biomedical Engineering 37(3), 586-594.

Richman, J.S., Moorman, J.R., 2000. Physiological time-series analysis using approximate entropy and sample entropy. Am J Physiol Heart Circ Physiol 278(6), H2039-2049.

Tzourio-Mazoyer, N., Landeau, B., Papathanassiou, D., Crivello, F., Etard, O., Delcroix, N., Mazoyer, B., Joliot, M., 2002. Automated anatomical labeling of activations in SPM using a macroscopic anatomical parcellation of the MNI MRI single-subject brain. Neuroimage 15(1), 273-289.

Xia, M., Wang, J., He, Y., 2013. BrainNet Viewer: a network visualization tool for human brain connectomics. PloS one 8(7), e68910.

Zhang, T., Yang, Z., Coote, J.H., 2007. Cross‐sample entropy statistic as a measure of complexity and regularity of renal sympathetic nerve activity in the rat. Experimental physiology 92(4), 659-669.
