# Supplementary material for: Automatic diagnosis of late-life depression by 3D convolutional neural networks and cross-sample Entropy analysis from resting-state fMRI
Source: Brain Imaging Behav. 2022 Nov 24;17(1):125–35. doi: 10.1007/s11682-022-00748-0 (PMC9922223; doi:10.1007/s11682-022-00748-0)
Supplement: Supplementary file 3 — Supplementary Material 3 [file 11682_2022_748_MOESM3_ESM.docx]

**Manuscript ID** number (*if available*): BIOR-D-22-00163

**Title**: **Automatic Diagnosis of Late-life Depression by 3D Convolutional Neural Networks and Cross-sample Entropy Analysis from Resting-state fMRI**

Name of **First** **Author**: Chemin Lin

**E-mail address** of First Author: chemin117@gmail.com

Name of **Corresponding** **Author**: Tatia Mei-Chun, Lee ; Shun-Chi Wu

**E-mail address** of Corresponding Author: [tmclee@hku.hk](mailto:tmclee@hku.hk) ; [shunchi.wu@mx.nthu.edu.tw](mailto:shunchi.wu@mx.nthu.edu.tw)

**AUTHORSHIP**

I, the undersigned author(s), certify that:

1. I have read and approved the final version of the manuscript.
2. I have made substantial contributions to the submitted work, which may include study design, data acquisition and/or analysis, and data interpretation.
3. I have made significant contributions to the preparation of the manuscript and/or critical revisions for important intellectual content.
4. I will be accountable for all aspects of the submitted work.
5. I agree to help investigate and resolve any issues/questions that may arise regarding the accuracy and integrity of the submitted work.

I understand that if necessary, the Editor-in-Chief of *Brain Imaging and Behavior* or designate may request deidentified data that has been submitted as part of the manuscript. In this event, I agree to produce the data.

I certify that this manuscript is not under consideration for publication in any other journal, nor has it been accepted for publication in any form, and no rights have been assigned to a third party.

I certify that all individuals who have made specific contributions to this manuscript but who do not fulfill the authorship criteria are listed with their specific contributions in the Acknowledgments section of the manuscript.

All funding sources directly or indirectly supporting this research have been acknowledged within the manuscript, including grant numbers where appropriate.

I understand that after the initial submission, authorship changes are strongly discouraged unless clearly warranted. If a major revision is submitted and authors have been added, removed or reordered, a rationale must be clearly indicated on the authorship change form, and all authors, prior and newly proposed, must sign the form indicating their agreement. Original signatures are required. Changes are subject to approval by the Editor-in-Chief.

**CONFLICT OF INTEREST AND FINANCIAL DISCLOSURE**

Within this manuscript, I agree to disclose any potential conflicts of interest, financial or otherwise, that might be perceived as influencing the objectivity of my work.

- Potential sources of conflict include but are not limited to patents, copyrights, royalties, or stock ownership, membership on a company’s board of directors, membership on a company’s advisory board, and consultancy or speaker’s fees from a company.

I understand that the conflict of interest section is to be placed at the end of the manuscript:

“None of the authors have a conflict of interest to declare.”

____________________________________________________________________________

By signing this document, I agree to accept full responsibility for the work submitted in the manuscript, including the accuracy and integrity of the data and data analyses.

Additionally, I assert that there are no conflicts of interest, either personal or institutional, that have compromised the integrity of the work reported in this manuscript.


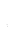


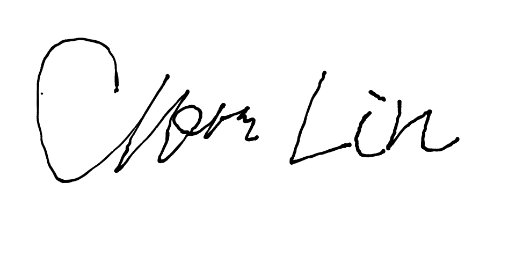

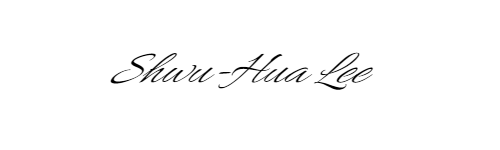


__________________________________ __________________________________

Chemin, Lin 2022/05/23 Shwu-Hua, Lee 2022/05/23


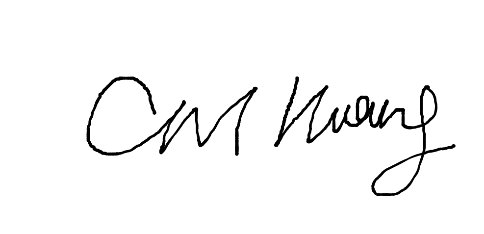

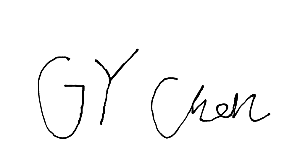


__________________________________ __________________________________

Chih-Mao, Huang 2022/05/23 Guan-Yen Chen 2022/05/23


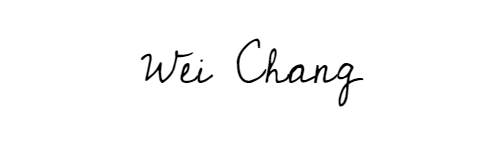

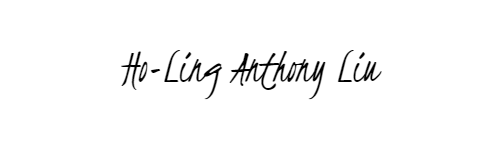


_________________________________ __________________________________

Wei Chang 2022/05/23 Ho-Ling Anthony, Liu 2022/05/23


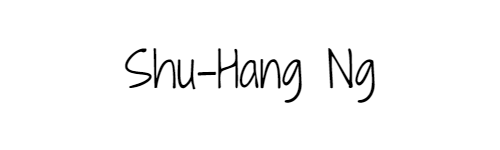

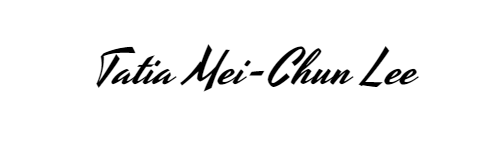


__________________________________ __________________________________

Shu-Hang Ng 2022/05/23 Tatia Mei-Chun, Lee 2022/05/23


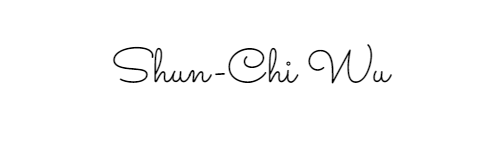


_____________________________

Shun-Chi Wu 2022/05/23
